# Distribution and sexual dimorphism of the crab *Xenograpsus testudinatus* from the hydrothermal vent field of Kueishan Island, northeastern Taiwan

**DOI:** 10.1371/journal.pone.0230742

**Published:** 2020-03-26

**Authors:** Li-Chun Tseng, Pin-Yi Yu, Jiang-Shiou Hwang

**Affiliations:** 1 Institute of Marine Biology, College of Life Sciences, National Taiwan Ocean University, Keelung, Taiwan; 2 Center of Excellence for the Oceans, National Taiwan Ocean University, Keelung, Taiwan; KAOHSIUNG MEDICAL UNIVERSITY, TAIWAN

## Abstract

The sulphur-rich and acidic vent waters of a shallow hydrothermal vent field next to Kueishan Island in Taiwan provide a specific and generally toxic environment. Among only a few aquatic organisms able to survive there, the grapsoid crab *Xenograpsus testudinatus* is the dominant species with a high population density in the vent area. Here we study the gender-specific distribution, morphological traits, and relationship of wet weight *vs*. carapace width of this crab. A total of 1120 individuals including 831 male and 289 female (included 15 ovigerous) were examined during August and September in 2011 and May and September in 2012. Except in August 2011, there are no significant differences in the distribution of *X*. *testudinatus* in the hydrothermal vent area from the vent spout during most months. Among crabs, the weight of male (6.87 ± 2.90 g) was significantly heavier than that of females (4.17 ± 1.25 g) (*p* < 0.001, Student’s t-test). As for the wet weight of crabs, significant differences were noted in both the length of chela and the width of carapace between males and females. Sexual dimorphism of *X*. *testudinatus* is evident in three morphological traits. Pearson’s correlation showed a significant and positive correlation (*p* < 0.001) of wet weight, width of carapace and length of chela of the two sexes. Ovigerous crabs (shortest carapace width: 1.93 cm) were present in the specimen collected from August 2011 and May 2012. The ovigerous crabs were not found in the samples collected from September in both years 2011 and 2012, indicating that reproduction may have ceased during the period of sampling. The present results suggested that the reproductive period of *X*. *testudinatus* was before September. The distribution pattern and sexual dimorphism of *X*. *testudinatus* provided a better understanding of the idiobiology of this dominant metazoan in the hydrothermal vent area.

## Introduction

Submarine hydrothermal vents are called shallow if their depth is above 200 meters in the range of the euphotic zone at many places worldwide. If their location in a depth range deeper than 200 meters in the aphotic zone is called deep sea hydrothermal vent. The primary productivity of the euphotic zone hydrothermal vent comes from photosynthetic and chemosynthetic organisms. The primary productivity within deep-sea hydrothermal vents comes only from chemoautotrophic prokaryotes. The fauna of the two regions are also significantly different [1]. Due to the fact that hydrothermal vent water has the characteristics of high temperature and acidification, shallow hydrothermal vent areas are a good place to study cutting-edge future ocean issues: acidification, warming, and climate change [2]. There is a small volcanic island located at the northeastern coast off of Taiwan [3], named Kueishan Island (also called as Kueishantao, Gueishan Island or Turtle Island). This island originated from volcanic eruptions about 7000 ± 700 years ago [4]. Due to the active magma beneath the earth's crust [5], Kueishan Island is defined as an active volcano in the Taiwan area [4,6]. Heated groundwater is ejected from the cracks in the seabed, creating a hydrothermal vent area in the east of the island [4,7]. Seabed temperature reveals that the heat flow at offshore Kueishan Island was about 700–1284 mW/m^2^, the highest record up to 2500 mW/m^2^ [7].

The groundwater erupting in the hydrothermal vent area is of particular characteristics, being of lowest pH (1.52) and highest temperature (116 ^o^C) [8–10]. The hydrothermal vent water is toxic because it is rich of sulfides among other natural compounds [11,12]. In such a special environment, a variety of highly adapted organisms occur, such as the crab *Macromedaeus distinguendus* (De Haan, 1835) [13] and *Xenograpsus testudinatus* N. K. Ng, J.-F. Huang & Ho, 2000 [14], copepods [15–16], anemones [17–18], several species of mollusks [17–20], and the azooxanthellate scleractinian coral *Tubastrea* spp. [17].

Among these macroinvertebrates, the most abundant and representative benthic invertebrate is the grapsoid crab *X*. *testudinatus*, inhabiting an open seabed of the hydrothermal vent area, with large rocks and sandy bottoms [11,21]. Specimens of this crab were firstly discovered from fishery bycatches [14]. Later there were several related research reports on it, such as on its detoxification mechanism [17], bioaccumulation of trace metals [22], metabolic energy demands, and food utilization [23], feeding [24–25], selective behavior [26], role in the trophic structure [27–28], and reproduction [28]. However, although this species has been exploited during two decades, the *in situ* distribution pattern and many aspects of biology of this crab in the hydrothermal vent area has not been understood.

In this study, the collection of specimen of this crab was carried out for several months by randomly dispatching crab traps at different distances from the hydrothermal vent nozzles. The present study was aiming to understand: (a) the distribution pattern of different genders of the crab *X*. *testudinatus*, the relationship between body size, and the distance to the hydrothermal vents, (b) the morphological characteristics, and (c) the possible breeding season from the records of ovigerous individuals.

## Material and methods

### Field sampling and sample treatment

**Fig 1:**
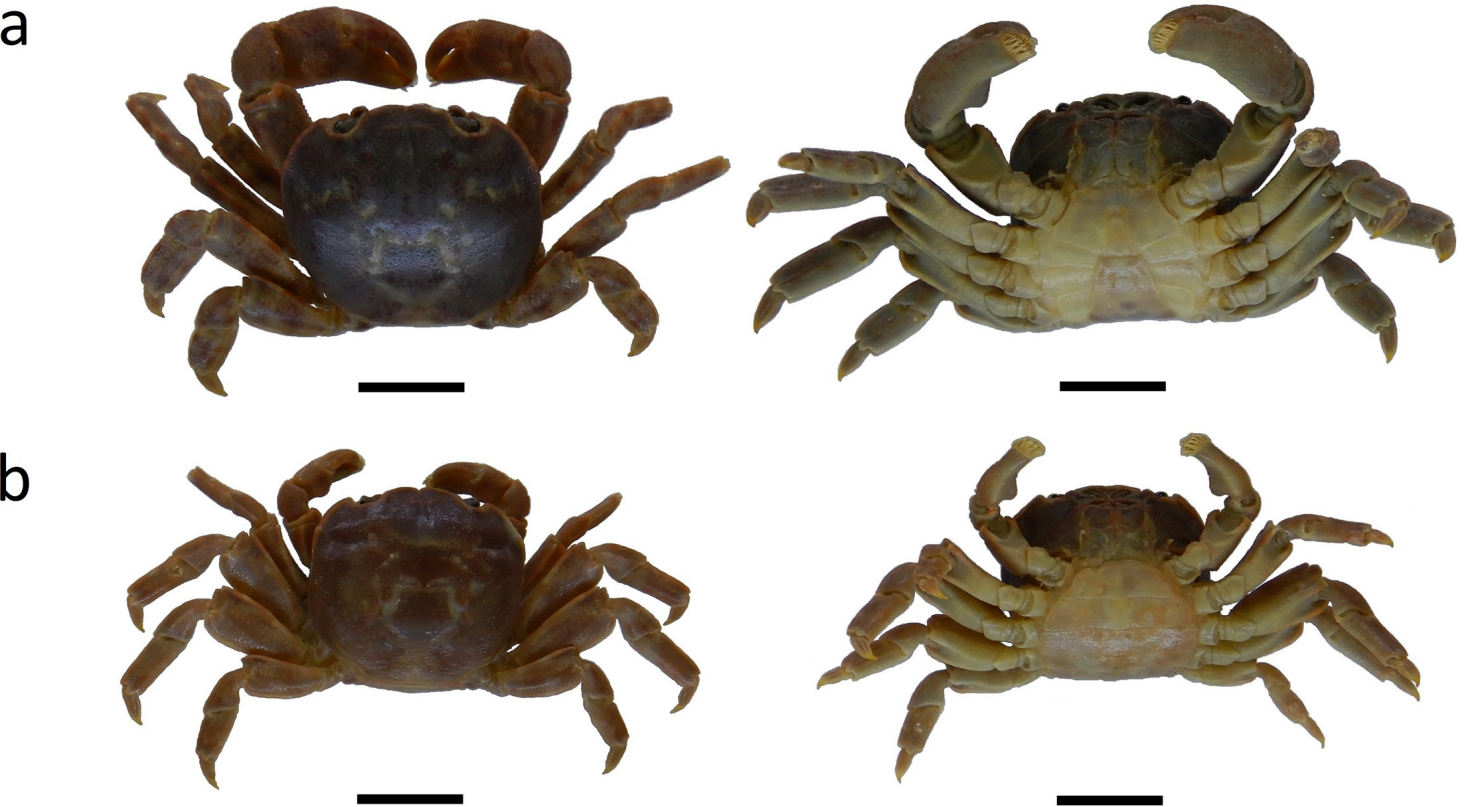
*Xenograpsus testudinatus* collected from the toxic shallow hydrothermal vent area in Kueishan Island. Male (a) and female (b) (left: dorsal view and right: ventral view). The scale bar represents 10 mm.

**Fig 2:**
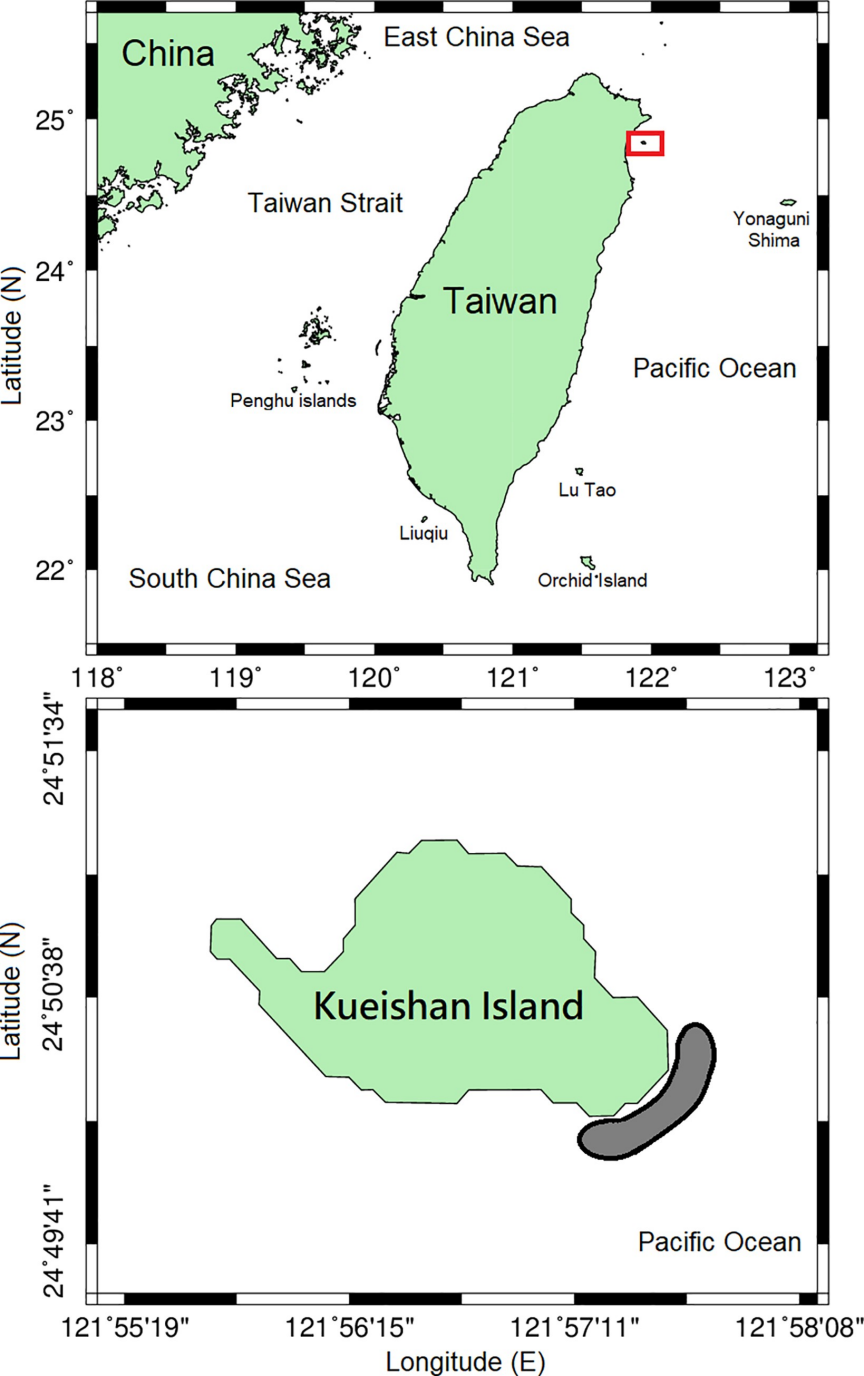
**Map of the study area (upper) and sampling locations (lower) around Kueishan Island during August 2011 to September in 2012.** The grey area indicates the sampling station location.

**Fig 3:**
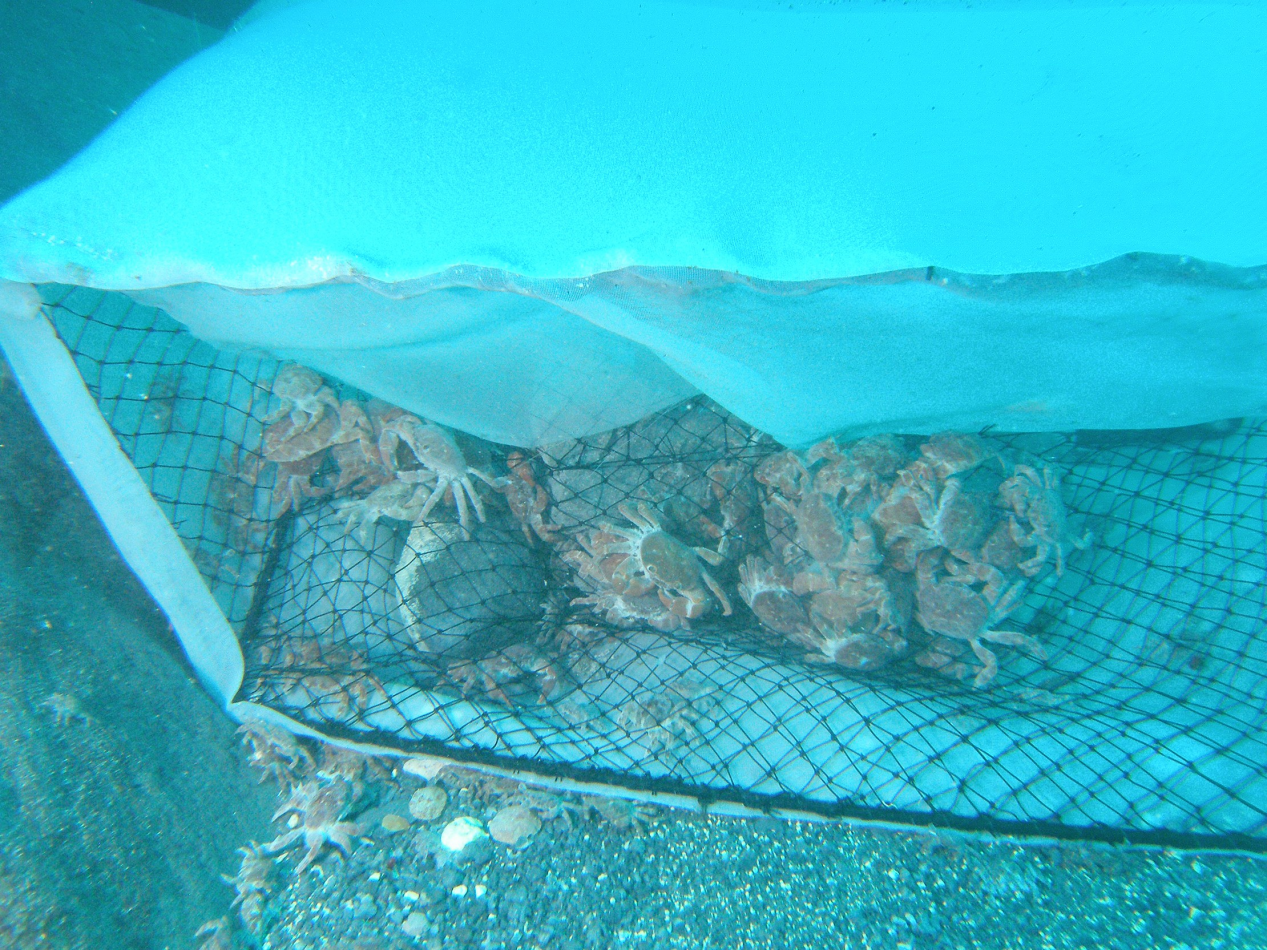
Photo of crabs and sampling trap.

The present study was conducted on board during August 14 and September 6 in 2011, and May 15 and September 11 in 2012. The study crabs *X*. *testudinatus* (Fig 1) were collected from the hydrothermal vent area of Kueishan Island off northeastern Taiwan (Fig 2) by SCUBA divers at a depth of 18–20 m. Rectangular crab traps (L × W × H = 60.5 × 43 × 21, mouth opening 43 × 21, unit: cm) were applied (Fig 3). The crab traps were wrapped with a mesh size of 333 μm zooplankton net to avoid the escape of small crabs. Three crab traps were setup on the seabed following a transect line to reveal the distribution patterns of crab populations in the vent area. The distances from the vent site to three crab traps are 5 m, 20 m and 35 m (± 1 m), respectively. The fresh fish meat of *Cololabis saira* (Brevoort, 1856) was tied at the bottom within the traps as bait to lure the crabs. The crab traps were withdrawn by SCUBA divers after a 2 hour collecting period. The specimens were rapidly placed into plastic bags and labeled, and further saved in iceboxes containing ice to preserve the specimens during their transportation to the laboratory.

### Body measurements

In the laboratory, samples were sorted and gender was identified by the abdominal structure (Fig 1), and females carrying eggs were classified as ovigerous. All crabs were measured according to three characteristics: wet body weight (g), width of carapace (cm), and length of chela (cm). The weight of crabs was measured by an analytical electronic microbalance (Type AG 135, Mettler Toledo, Switzerland), width of carapace and length of the chela of the right claw were measured with digimatic calipers (Type CD-6'' CSX, Mitutoyo, Japan). The values of carapace width and weight were separated for sex, and subsequently applied to estimate the relationship between carapace width and weight by the following exponential equation [29]:

$$W=aL^{b}$$ (1)

where W = wet weight of crab, L = width of carapace, *a* = y-intercept or initial growth coefficient, and *b* = slope or growth coefficient. Constants *a* and *b* were calculated using a simple exponential regression method.

### Statistical analysis

In order to reveal the abundance and distribution of crabs among three distances, we applied one-way ANOVA with post-hoc Tukey's Honest Significant Difference (HSD) test. A univariate method of the general linear model with post-hoc Tukey's HSD test was applied to test the effects between sampling month and sampling location on the body weight of crabs. Further, the relationship between body weight with width of carapace, and with length of chela was studied by using the Pearson’s product moment correlation. To identify differences in three characterizes between two genders of crabs, Student's t-test was applied. Statistical analysis was done by using the statistics package SPSS v.24.

## Results

### Crab specimen in the present study

**Fig 4:**
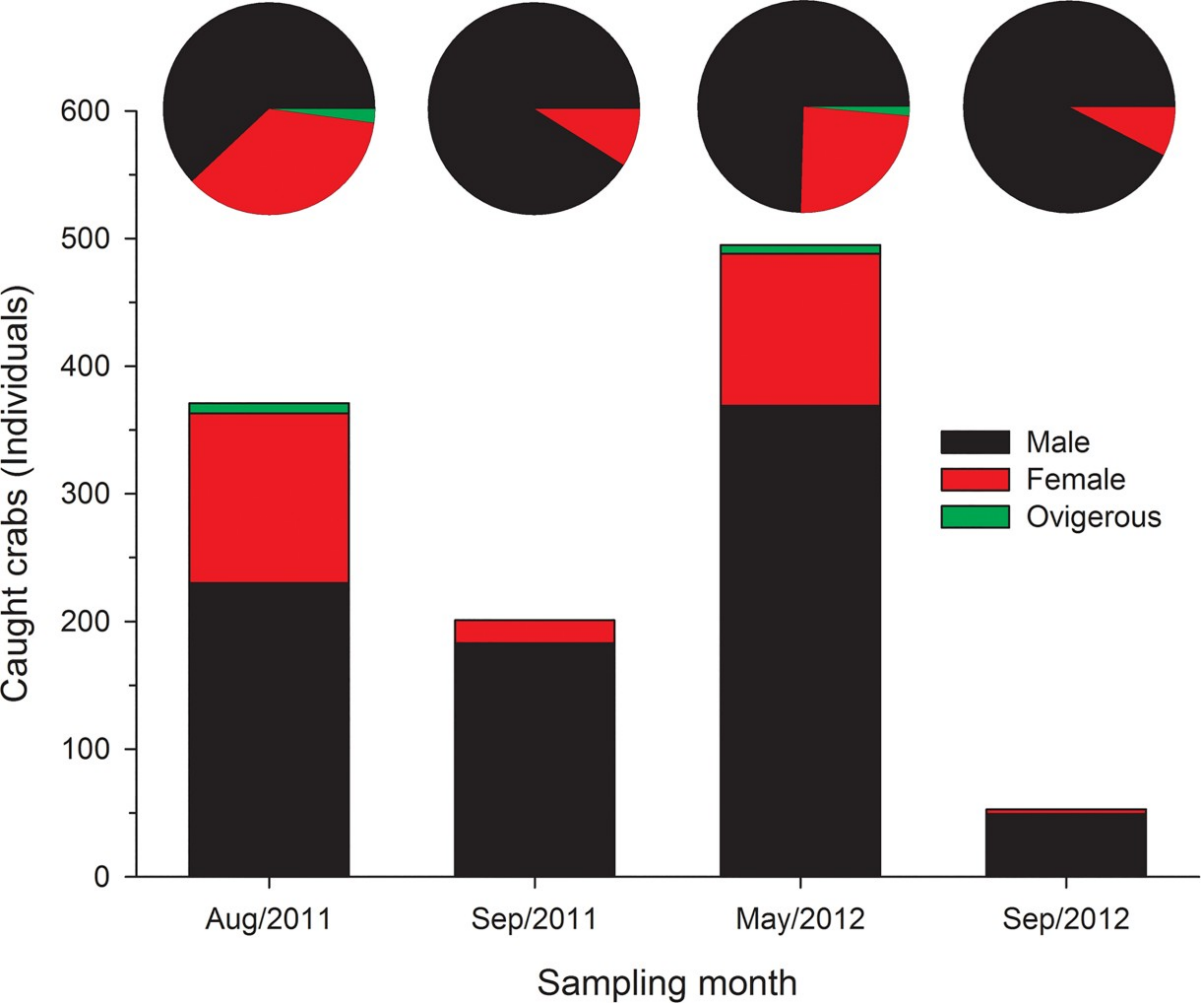
Number of crabs *Xenograpsus testudinatus* and proportion of male, female and ovigerous collected from different sampling months.

A total of 1120 crabs were harvested from the vent site during 4 sampling periods: 371 individuals on August 14, 2011; 201 individuals on September 6, 2011; 495 individuals on May 15, 2012; and 53 individuals on September 11, 2012 (Fig 4). Among the crab of all samples, the numerically dominating crabs were male (831 individuals (inds.), relative abundance, RA: 74.2%). Remaining female (274 inds., RA: 24.46%) and ovigerous crab (15 inds., RA: 1.34%), their abundance comprised of 25.8% of the overall crab counts. The abundance and proportions of crabs in 4 sampling periods varied. No ovigerous female crabs were identified from specimens collected in September of both years 2011 and 2012 (Fig 4). The sex ratio of male:female was 61.99:38.01, 91.04:8.96, 74.55:25.45 and 92.45:7.55 on August 14, 2011, on September 6, 2011, on May 15, 2012, on September 11, 2012, respectively. Male crabs were more abundant than females; and dominant during the 4 sampling months.

### Distribution of gender in crab populations

**Fig 5:**
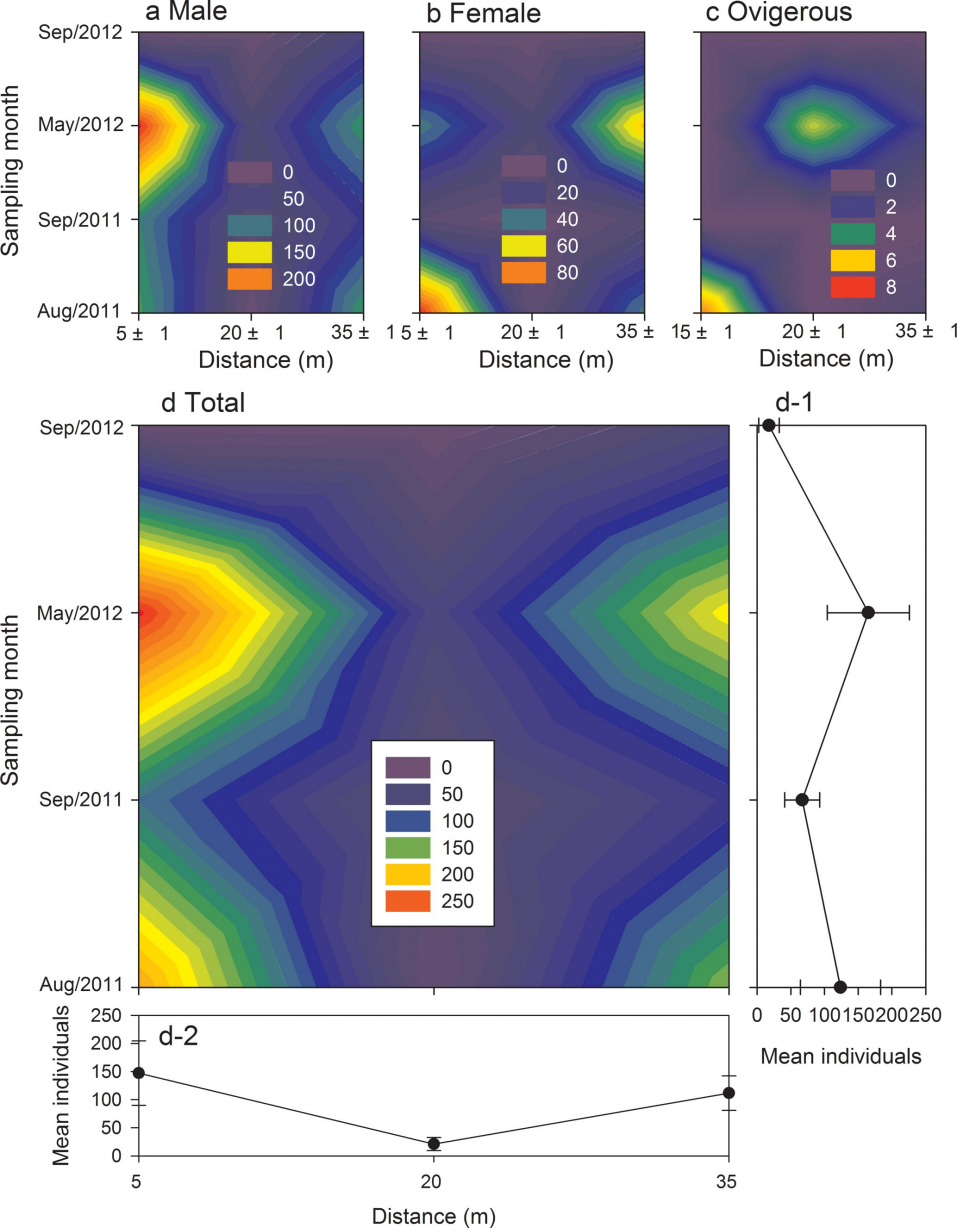
Temporal and spatial variation in abundance (individuals) of male (a), female (b), ovigerous (c), total (d) of crabs *Xenograpsus testudinatus* among four sampling months from August 2011 to September 2011 and crab collected from different distance locations.

The distribution of *X*. *testudinatus* of both genders showed clear changing pattern in different sampling months (Fig 5). The highest number of males was recorded at a distance of 5(±1) meters (225 individuals) from the hydrothermal vent spout in May 2012 (Fig 5A), Females were gathered at 5(±1) meters (90 individuals) and 35(±1) meters (66 individuals) in August 2011 and May 2015, respectively (Fig 5B). The higher record of ovigerous female distribution was at 5(±1) meters (7 individuals) and 20(±1) meters (5 individuals) in August 2011 and May 2015, respectively (Fig 5C). Overall, the distribution of total crabs was relatively high at locations 5(±1) meters and 35(±1) meters, whereas the number at 20(±1) meters was low (Fig 5D).

### Distribution patterns of crabs

**Fig 6:**
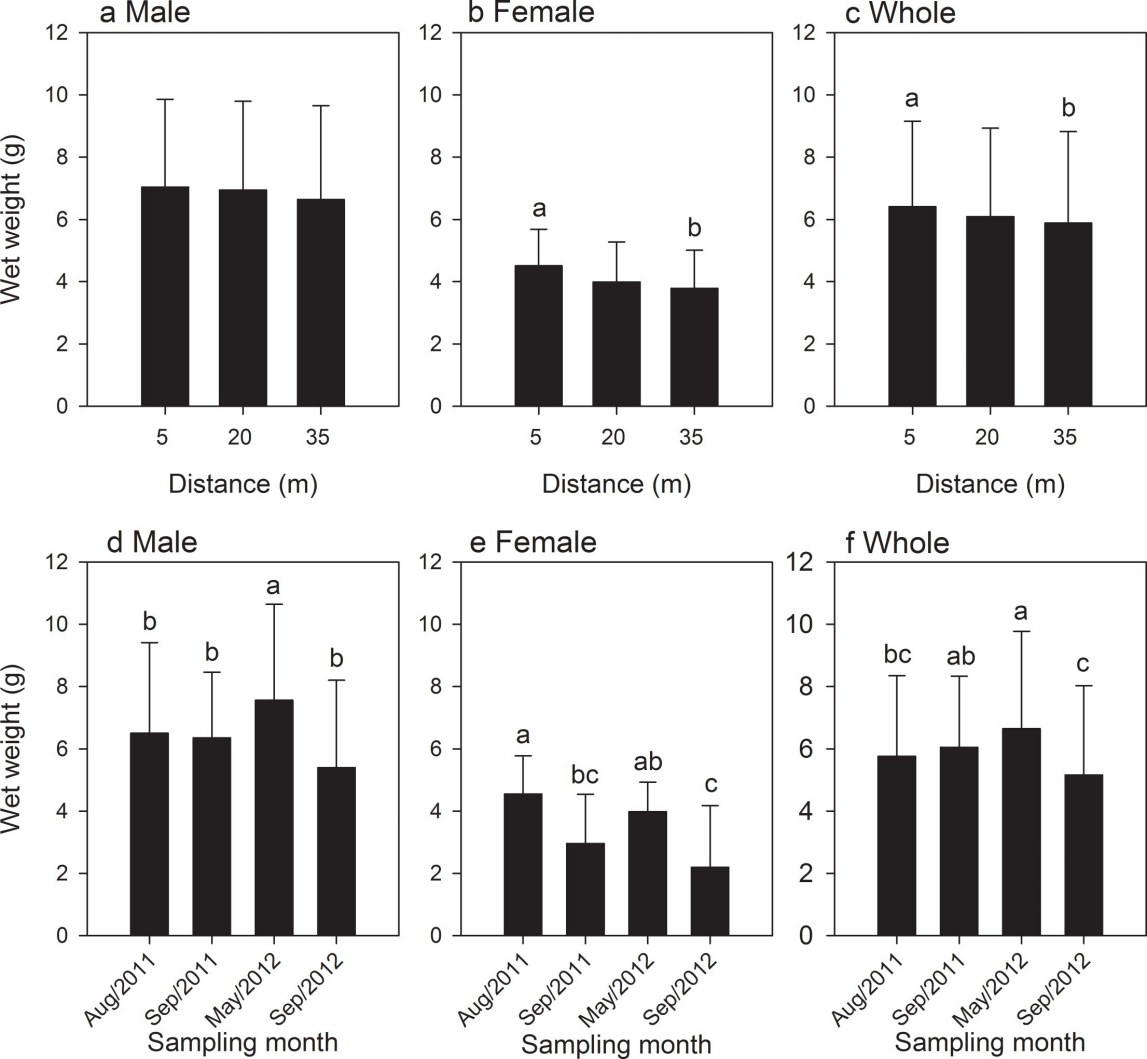
Statistical results of the wet weight of male (a), female (b) and whole crabs (c) of *Xenograpsus testudinatus* with a different sampling distance; as well as male (d), female (e) and whole crabs (f) of *X*. *testudinatus* with a different sampling month; the various superscripts indicate the significant differences (*p* < 0.05, one-way ANOVA) among the sampling locations.

In both sexes of *X*. *testudinatus*, a significantly positive correlation among three morphological traits was detected for crabs (*p* < 0.001 in all cases, Pearson's correlation, Table 1). The slope of the relationship between carapace width versus wet weight was lower in males (r = 0.874) than in females (r = 0.904). The same correlation occurred between carapace width *versus* chela length in the males (r = 0.672) and lower than in females (r = 0.840). In contrast, the relationship between chela length versus wet weight was higher in males (r = 0.913) than in females (r = 0.685). Based on the results of Pearson's correlation (Table 1), we used body weight of the crabs as an indicator for the distribution of crabs in the different distances from the sampling location, and among different sampling months. The results of one-way ANOVA showed that body weight of male and female *X*. *testudinatus* crabs were significantly higher at locations 5 ± 1 (m) and 35 ± 1 (m) than the 20 ± 1 (m) in Aug/2011; there are no significant differences in body size in the other sampling months (Table 2). The distribution of both sexes did not show a clear pattern the body size fraction. Furthermore, the results of two-way ANOVA found that the wet weight (body size) of males, females, and whole crabs show significant differences in distance, month, and interaction effects (*p* < 0.05, ANOVA; Table 3, Fig 6).

**Table 1:** Correlation results of body weight (g), carapace width (cm), and chela length (cm) of male and female *Xenograpsus testudinatus* according to pearson’s correlation analysis.

		Male (n = 831)		
		Wet weight	Carapace width	Chela length
Female (n = 289)	Wet weight		0.874** (< 0.001)	0.913** (< 0.001)
	Carapace width	0.904** (< 0.001)		0.840** (< 0.001)
	Chela length	0.685** (< 0.001)	0.672** (< 0.001)	

The value represents the correlation (r), and the value in the parentheses represents *p*

*Significant at *p* < 0.05 (2-tailed)

**significant at *p* < 0.01 (2-tailed).

**Table 2:** Body weight (g) (mean ± standard variation) of male, female and ovigerous *Xenograpsus testudinatus*, and the statistical results of one-way ANOVA (between groups) of three sampling distances.

	Distance (m)			One-way ANOVA
	5 ± 1	20 ± 1	35 ± 1	
Male				
Aug/2011	6.66 ± 3.11 ^a^	2.29 ± 2.04 ^b^	6.52 ± 2.6 ^a^	*p* = 0.04*
Sep/2011	6.33 ± 2.18	6.47 ± 1.45	6.35 ± 2.2	*p* = 0.966
May/2012	7.53 ± 2.85	7.87 ± 2.9	7.53 ± 3.59	*p* = 0.862
Sep/2012	7.55 ± 2.27	N.F.	5.21 ± 2.79	*p* = 0.112
Female				
Aug/2011	4.81 ± 1.17 ^a^	2.54 ± 2.01 ^c^	4.06 ± 0.86 ^b^	*p* < 0.001**
Sep/2011	3.56 ± 0.61	N.F.	2.48 ± 1.96	*p* = 0.154
May/2012	3.96 ± 0.77	4.22 ± 0.61	3.87 ± 1.1	*p* = 0.445
Sep/2012	1.33	N.F.	2.47 ± 2.34	*p* = 0.714
Ovigerous				
Aug/2011	5.39 ± 1.07	N.F.	4.31	*p* = 0.381
Sep/2011	N.F.	N.F.	N.F.	*-*
May/2012	N.F.	4.77 ± 0.74	4.15 ± 0.28	*p* = 0.322
Sep/2012	N.F.	N.F.	N.F.	*-*

N.F. = not found

* Significant at the *p* < 0.05 level (2-tailed)

** significant at the *p* < 0.01 level (2-tailed); the various superscripts indicate the significant differences (*p* < 0.05, one-way ANOVA) among the sampling locations.

**Table 3:** Statistical results of two-way ANOVA of the wet weight of two genders and whole crabs of *Xenograpsus testudinatus* with a different sampling distances and different sampling months.

Source	S.S	d.f.	M.S.	F-value	*p* -value
Male					
Distance	69.42	2	34.71	4.37	0.013*
Month	276.85	3	92.28	11.62	< 0.001**
Distance × Month	113.09	5	22.62	2.85	0.015*
Error	6509.91	820	7.94		
Female					
Distance	8.35	2	4.18	3.51	0.031*
Month	38.21	3	12.74	10.70	< 0.001**
Distance × Month	31.00	4	7.75	6.51	< 0.001**
Error	332.02	279	1.19		
Whole crabs					
Distance	68.86	2	34.43	4.46	0.012*
Month	232.81	3	77.60	10.05	< 0.001**
Distance × Month	156.54	5	31.31	4.06	0.001**
Error	8559.72	1109	7.72		

The values of S.S. = type III sum of squares, d.f. = degree of freedom, M.S. = mean square.

* Significant at the *p* < 0.05 level (2-tailed)

** significant at the *p* < 0.01 level (2-tailed).

### Morphological characteristics

**Fig 7:**
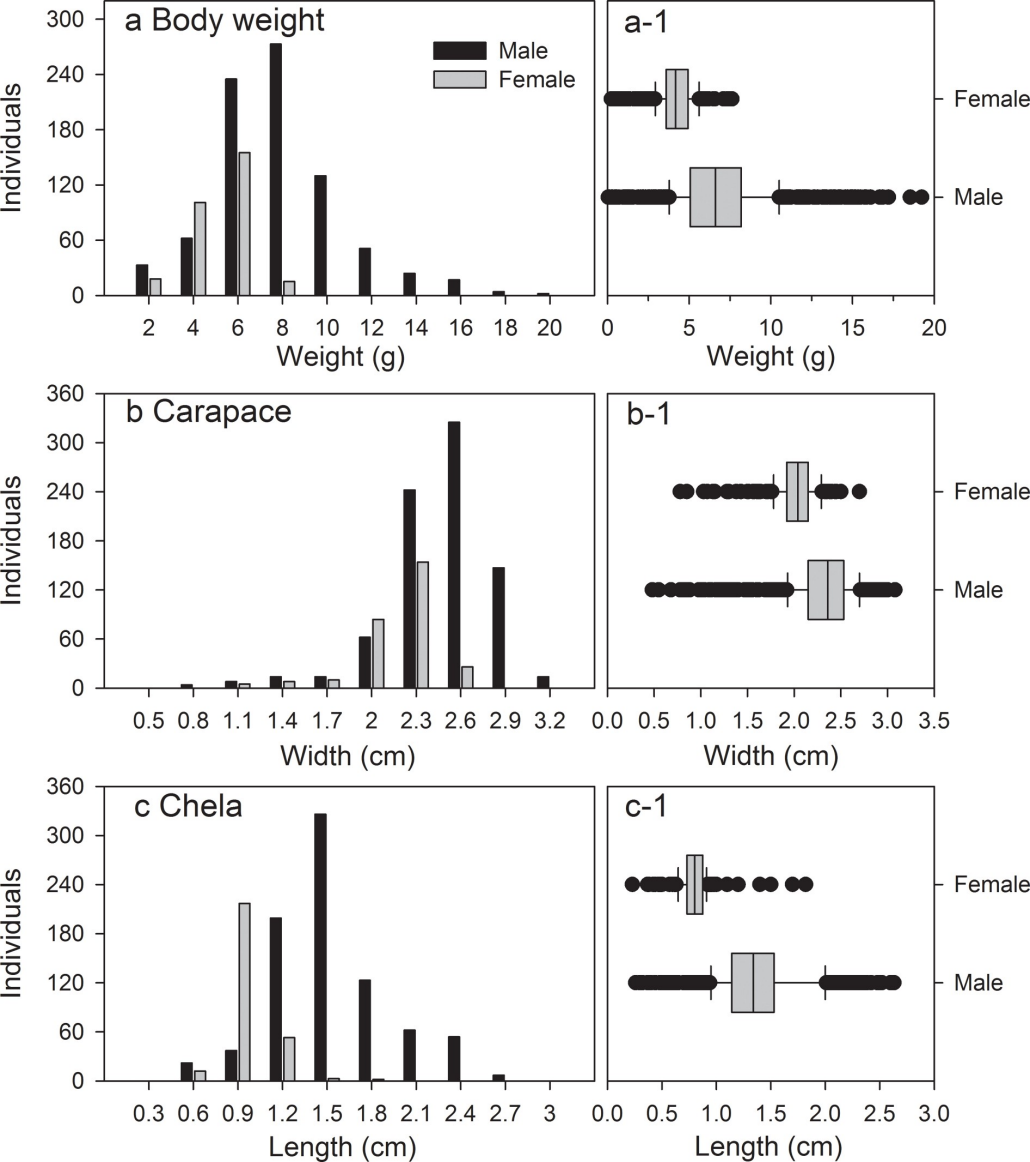
A survey of the distribution of female and male morphological measurements for the all crabs, wet body weight (a), carapace width (b), and chela length (c).

The ranges and mean (± standard deviation) of individual weight (g), carapace width (cm), and length of the chela (cm) of both sexes of *X*. *testudinatus* are shown in Fig 7. The wet weight of male and female *X*. *testudinatus* was 0.06–19.23 (6.87 ± 2.90) and 0.24–7.26 (4.17 ± 1.25) g, respectively. The difference between the wet weight of males and females was significant (T = 21.73, df = 1073.5, *p* < 0.001, Student's t-test) (Fig 7A). The carapace width of male and female *X*. *testudinatus* was 0.48–3.08 (2.31 ± 0.36) and 0.78–2.70 (2.00 ± 0.27) cm, respectively. The carapace width (T = 15.30, df = 675.5, *p* < 0.001, Student's t-test) were significantly different in both sexes (Fig 7B). In addition, the shortest carapace width of ovigerous females was 1.93 cm, which indicates that female crabs of this size have gained sexual maturity. The chela length of male and female *X*. *testudinatus* ranged between 0.26–2.63 (1.37 ± 0.40) and 0.23–1.82 (0.80 ± 0.16) cm, respectively. The difference of the chela length between males and females was significant (T = 34.07, df = 1102.8, *p* < 0.001, Student's t-test) (Fig 7C). The morphological characteristic indicated sexual dimorphism of *X*. *testudinatus*.

**Fig 8:**
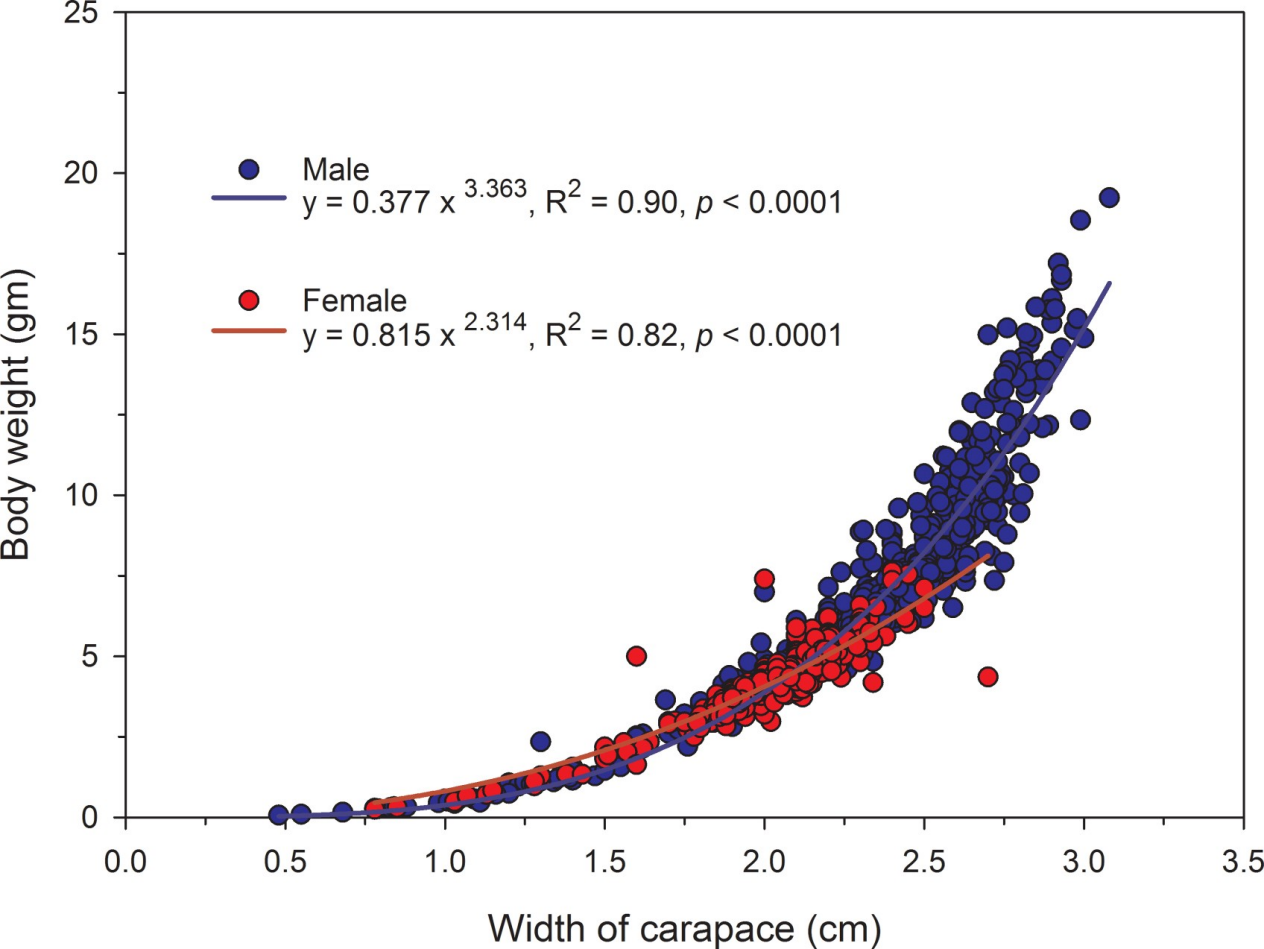
Relationship between the carapace width and wet weight of male and female *Xenograpsus testudinatus* collected from the hydrothermal vent area in Kueishan Island.

In this study we analyzed the biological traits of *X*. *testudinatus* at the vent site, and found that all morphological characteristics (wet weight, carapace width, and chela length) were significantly different in both sexes (*p* < 0.001, Pearson’s correlation analysis; Table 1). The ratio of wet weight:carapace width was significantly correlated in males (2) and females (3) (Fig 8) and was determined by the following functions:

$$y=0.377\;x^{3.363}\;\left(R^{2}=0.90,p<0.0001\right)$$ (2)

$$y=0.815\;x^{2.314}\;\left(R^{2}=0.82,p<0.0001\right)$$ (3)

where *y* = wet body weight (g) and *x* = carapace width (cm).

The growth rate of *X*. *testudinatus* females was slightly higher than the males before the carapace width of 2.07 cm, but the difference was not significant (Fig 8). Beyond 2.07cm, the growth rate of males was significantly higher than that of females, and the highest weight was 19.23 g in a male.

## Discussion

### Crab distribution in the hydrothermal vent area

Previous studies showed that hydrothermal vents are the most important habitat factor for the vent crab *X*. *testudinatus*. This crab could not be found otherwise along the shallow coast of Kueishan Island outside the hydrothermal vent area [18]. The main reason might be the acidity and sulfide toxicity of hydrothermal vent waters that made it impossible for the predators of *X*. *testudinatus* living in hydrothermal vent field for extended times. Secondly, since *X*. *testudinatus* developed a detoxification mechanism that helps them to adapt to this extreme environment [17]. Therefore, they show substantial reproduction in the hydrothermal vents area, and their population density was estimated to be 364 crabs per square meter (average density) [11].

A study used the laboratory acclimated wild-caught *X*. *testudinatus* to test their selective behaviors, pointing out that the crabs have a high probability of selection for environmental factors in the area of hydrothermal vents [26]. The preferences of these crabs may be due to the effects of growth, and preference for various environmental factors experienced during growth. Hung et al. [12] revealed that ovigerous females of *X*. *testudinatus* may migrate from regions of near the vent spout to regions of the vent-periphery to release their larvae, to avoid the larvae contacting highly toxic hydrothermal vent water. Such migration behavior also affects the distribution of crab populations. The present *in situ* study showed that there is no significant difference in the distribution of *X*. *testudinatus* in the hydrothermal vent area from the vent spout in most months (except for August 2011, Table 2). The main reason is that the sampling locations of the study were all in the hydrothermal vent area. Kueishan Island contained 12 intensively active hydrothermal vents and 15 quiet hydrothermal vents within an area of 1.3 square kilometers in the shallow hydrothermal vent area [8]. The direction of the current is constantly changing due to the influence of tides and winds. Therefore, the boundary of the hydrothermal vent waters is fluctuating over time. The affected area is wider than the actual distribution of the hydrothermal vent spouts. The most distant sampling locations in the present study are 35 (± 1) meters from the hydrothermal vent spout. These locations are still in the hydrothermal vent area. We found that there is no significant difference in the distribution of crabs among sampling locations at different distances.

Furthermore, food availability is determinant of population density and distribution [30]. Toxic hydrothermal vent water in the tidal transition period will kill zooplankton transported by the current entering the hydrothermal vent area, the vent crabs *X*. *testudinatus* also feeds on marine snow formed by dead zooplankton [11]. Studies reported that the crabs *X*. *testudinatus* is omnivores and scavengers, feeding on diverse food from microorganisms to various organic detritus [24,27]. The sulfur-rich sediments and vent water contained high diversity of bacterial communities and chemoautotrophic populations in the hydrothermal vent area [31,32]. There is no difference in the distribution of aforementioned foods in the hydrothermal vent area, so there is no significant difference in the spatial distribution of *X*. *testudinatus* in the area.

Habitat selection is the result of long-term evolution and adaptation [33–35]. Intraspecific competition determines the extent of habitat expansion [36]. There is another crab *Macromedaeus distinguendus* (De Haan, 1835) (Brachyuran: Xanthid) in the hydrothermal vent area, and its size of population is very small [13]. Unlike *X*. *testudinatus*, this crab (*M*. *distinguendus*) can also be found in the waters outside the hydrothermal vent area [37]. From the geographical distribution of the two crab species, the population of *X*. *testudinatus* cannot be extended beyond the hydrothermal vent area. The *M*. *distinguendus* may slowly evolve detoxification mechanisms against toxic hydrothermal vent waters that expand its habitat into this area. On the other hand, *Xenograpsus testudinatus* highly rely on toxic hydrothermal vent waters [18], accordingly their population size and distribution range are inevitably regulated by the hydrothermal vent [38]. Recent reports indicate that the hydrothermal vent area of Kueishan Island was affected by earthquakes, heavy precipitation and typhoons, causing the collapse and were partially buried [39,40]. Such environmental changes may further reduce the population size and distribution range of *X*. *testudinatus* [34,35].

### Morphological traits, allometrical growth and sexual dimorphism

The body size of male *X*. *testudinatus* is relatively larger than in females. A larger size also means being more robust, this can provide protection against environmental stress and resources competition [41]. Lee [42] reported that the evolutionary complex morphological structures of brachyuran crustaceans provide responses to the sexual selection under a combination of three selection pressures: foraging behaviour, reproduction, and agonistic behavior. This study found that male *X*. *testudinatus* have larger chelipeds. This may be important for provisioning females for copulation and also cause more damage during fights against other males [43].

The present study found that slope value of male and female crab was 3.363 and 2.314 respectively, indicating that the growth pattern of the *X*. *testudinatus* was allometrically positive [44]. The growth rate of both sexes has a clear change between the young and the adult stages (Fig 6). The gender differences are not significant during the young stages, but only in mature individuals. The changes in growth rate may be related to evolutionary adaptations [42]. Simpson et al. [45] suggested that environmental conditions and challenges encountered by individuals of two genders in younger stages are similar, so the rate of growth shows the same trend; adult males are larger in size, which contribute to interspecies competition for food, habitat and mating expediency [41,43,46], therefore the rate of growth increases faster than in females. We found in the present study that beyond a carapace width of 2.07 cm, male individuals get grew faster than in females (Fig 8). We found that the shortest carapace width of an ovigerous crab was 1.93 cm, indicating that the female individual could start breeding from this size. We assume that the growth rate of females slowed down with the onset of reproductive and parental investment. Since male crabs investment in reproduction by sperm production is much less, its resources can be used for individual growth, therefore beyond carapace width of 2.07 cm; the growth rate is relatively fast. Pinheiro and Hattori [47] reported that morphometric allometric studies of *Uca cordatus* (Linnaeus, 1763) found a higher ratio of chela growth for adult males than in females, indicating potential utilization during reproductive behavior. In addition, the growth coefficient (slope) of *X*. *testudinatus* is bigger than in the *M*. *distinguendus* (male: 1.237, female: 1.222) in the same hydrothermal vent area [13]. However, the relationship pattern of wet weight *vs*. carapace width can be affected by various biological and non-biological factors, such as growth pattern, quality and quantity of foods, season and environmental conditions [48], as well as physical factors such as salinity, temperature, pH, rainfall, and dissolved oxygen [49].

The variation of three morphological traits of *X*. *testudinatus* indicated that the shape difference of both genders was due to sexual dimorphism. Sexual dimorphism is commonly found in crabs [43,50–54]. In brachyurans, sexual dimorphism is common for females to have larger and wider abdomina than males; because they carry and incubate embryos attached to the abdominal appendages [55]. The present study found that *X*. *testudinatus* in the hydrothermal vent area had a clear pattern of sexual dimorphism by developing larger chelipeds in males, which is similar to fiddler crabs (*Uca* spp.) [43,51,56], and another hydrothermal vent crab *M*. *distinguendus* [13].

### Ovigerous state and breeding

The record in present study found that the number of ovigerous females was low, perhaps related to the adaptive behavior of ovigerous crab. Previous studies have shown that ovigerous crabs reduced the frequency of feeding and the number of molts during carrying eggs. Ovigerous crabs are preferred to hide in cryptic habits to avoid encounter of predators; reducing the frequency of decladding can avoid the loss of eggs during hatching, allowing female crabs to protect the eggs to release of crab larvae [57]. In addition, ovigerous crabs of *X*. *testudinatus* select places farther away the hydrothermal vent spout to release larvae, avoid highly toxic vent water kill larvae after hatched [28]. The above literatures explain why the proportion of ovigerous crabs caught in the hydrothermal vent area was low.

The ovigerous crabs of *X*. *testudinatus* were recorded in the August/2011 and May/2012 samples, and none found in the samples collected from September in both years 2011 and 2012; thus estimated that the reproductive period of *X*. *testudinatus* is before September. September is the transition period in northwest Pacific, the wind from southwest monsoon converted into the northeast monsoon. During the northeast monsoon period, the water temperature will decrease and the density of zooplankton decline in the waters around Kueishan Island [58]. *Xenograpsus testudinatus* to release larvae when the water temperature is warm; and high zooplankton density can provide adequate food for larvae.

## Conclusion

The present study provides the first documentation of the distribution, morphological traits, sexual dimorphism, and allometric growth in males and females of *X*. *testudinatus* from the shallow hydrothermal vent, Kueishan Island. The morphological differences between males and females reveal the evolution of *X*. *testudinatus* adapting to this particular environment. So far, studies on toxicity factors of hydrothermal vent waters affecting vent crabs, and courtship and mating behavior of this crab has not been understood. Further research is needed here and could contribute to understand the reproductive physiological adaptation associated with the male body differences of *X*. *testudinatus*.

## Supporting information

S1 File Supporting information file provides information of all figures. (XLSX)
